# Risk factors for community-acquired bacterial infection among young infants in South Asia: a longitudinal cohort study with nested case–control analysis

**DOI:** 10.1136/bmjgh-2022-009706

**Published:** 2022-11-01

**Authors:** Nicholas E Connor, Mohammad Shahidul Islam, Luke C Mullany, Nong Shang, Zulfiqar A Bhutta, Anita K M Zaidi, Sajid Soofi, Imran Nisar, Pinaki Panigrahi, Kalpana Panigrahi, Radhanath Satpathy, Anuradha Bose, Rita Isaac, Abdullah H Baqui, Dipak K Mitra, Qazi Sadeq-ur Rahman, Tanvir Hossain, Stephanie J Schrag, Jonas M Winchell, Melissa L Arvay, Maureen H Diaz, Jessica L Waller, Martin W Weber, Davidson H Hamer, Patricia Hibberd, A S M Nawshad Uddin Ahmed, Maksuda Islam, Mohammad Belal Hossain, Shamim A Qazi, Shams El Arifeen, Gary L Darmstadt, Samir K Saha

**Affiliations:** 1 Department of Microbiology, Child Health Research Foundation, Dhaka, Bangladesh; 2 Department of Clinical Research, London School of Hygiene & Tropical Medicine, London, UK; 3 Department of International Health, Johns Hopkins Bloomberg School of Public Health, Baltimore, Maryland, USA; 4 Division of Bacterial Diseases, Centers for Disease Control and Prevention, Atlanta, Georgia, USA; 5 Centre for Global Child Health, The Hospital for Sick Children, Toronto, Ontario, Canada; 6 Institute for Global Health and Development, The Aga Khan University, Karachi, Pakistan; 7 Department of Pediatrics and Child Health, The Aga Khan University, Karachi, Pakistan; 8 Department of Pediatrics, Georgetown University Medical Center, Washington, DC, USA; 9 AIPH University, Bhubaneswar, India; 10 Christian Medical College, Vellore, India; 11 Department of Public Health, North South University, Dhaka, Bangladesh; 12 International Centre for Diarrhoeal Disease Research, Bangladesh, Dhaka, Bangladesh; 13 Child and Adolescent Health and Development Division, WHO Regional Office for Europe, Copenhagen, Denmark; 14 Department of Global Health, Boston University School of Public Health, Boston, Massachusetts, USA; 15 Section of Infectious Diseases, Department of Medicine, Boston University School of Medicine, Boston, Massachusetts, USA; 16 Consultant and Researcher, (Retired WHO staff), Geneva, Switzerland; 17 Department of Pediatrics, Stanford University School of Medicine, Stanford, California, USA

**Keywords:** infections, diseases, disorders, injuries, cohort study, paediatrics, child health

## Abstract

**Objective:**

Risk factors predisposing infants to community-acquired bacterial infections during the first 2 months of life are poorly understood in South Asia. Identifying risk factors for infection could lead to improved preventive measures and antibiotic stewardship.

**Methods:**

Five sites in Bangladesh, India and Pakistan enrolled mother–child pairs via population-based pregnancy surveillance by community health workers. Medical, sociodemographic and epidemiological risk factor data were collected. Young infants aged 0–59 days with signs of possible serious bacterial infection (pSBI) and age-matched controls provided blood and respiratory specimens that were analysed by blood culture and real-time PCR. These tests were used to build a Bayesian partial latent class model (PLCM) capable of attributing the probable cause of each infant’s infection in the ANISA study. The collected risk factors from all mother–child pairs were classified and analysed against the PLCM using bivariate and stepwise logistic multivariable regression modelling to determine risk factors of probable bacterial infection.

**Results:**

Among 63 114 infants born, 14 655 were assessed and 6022 had signs of pSBI; of these, 81% (4859) provided blood samples for culture, 71% (4216) provided blood samples for quantitative PCR (qPCR) and 86% (5209) provided respiratory qPCR samples. Risk factors associated with bacterial-attributed infections included: low (relative risk (RR) 1.73, 95% credible interval (CrI) 1.42 to 2.11) and very low birth weight (RR 5.77, 95% CrI 3.73 to 8.94), male sex (RR 1.27, 95% CrI 1.07 to 1.52), breathing problems at birth (RR 2.50, 95% CrI 1.96 to 3.18), premature rupture of membranes (PROMs) (RR 1.27, 95% CrI 1.03 to 1.58) and being in the lowest three socioeconomic status quintiles (first RR 1.52, 95% CrI 1.07 to 2.16; second RR 1.41, 95% CrI 1.00 to 1.97; third RR 1.42, 95% CrI 1.01 to 1.99).

**Conclusion:**

Distinct risk factors: birth weight, male sex, breathing problems at birth and PROM were significantly associated with the development of bacterial sepsis across South Asian community settings, supporting refined clinical discernment and targeted use of antimicrobials.

**WHAT IS ALREADY KNOWN ON THIS TOPIC**

- Several different risk factors for neonatal sepsis—determined from a constellation of symptoms in hospitalised infants often lacking access to modern diagnostic techniques in low resource settings—have been used in management guidelines for all sepsis arising in community settings.

**WHAT THIS STUDY ADDS**

- This study analyses the risk factors associated with community-acquired young infant infections with bacterial aetiologies in five sites across three South Asian countries. These risk factors were used to construct multivariate models using stepwise regression for community-acquired bacterial infections by directly using a partial latent class model output derived from advanced laboratory diagnostics identifying aetiologies, building a common risk model for community-acquired neonatal sepsis across South Asia.

**HOW THIS STUDY MIGHT AFFECT RESEARCH, PRACTICE OR POLICY**

- Given this study of the risk factors associated with bacterial infections in young infants among a heterogeneous community field sites in South Asia, these data and findings may factor into refinement of point-of-care risk scoring algorithms, improvement of traditional treatment algorithms and, if further validated, be used to provide appropriate and judicious use of antibiotics.

## Introduction

Despite coordinated efforts to reduce child mortality over the past decade, deaths due to neonatal infections remain a major contributor to under-five mortality. With recent advances in other areas of child health, neonatal deaths comprise an increasing proportion of under-five mortality.^1–3^ Neonatal sepsis, pneumonia and meningitis have been estimated to cause a quarter of all newborn deaths,^4^ and this proportion might be even higher.^5^ South Asia and sub-Saharan Africa have the greatest burden of neonatal sepsis in the world.^6^ The Alliance for Maternal and Newborn Health Improvement (AMANHI) study found severe neonatal infections to be the second leading cause of neonatal deaths after perinatal asphyxia in South Asia (35%, 34–36) and sub-Saharan Africa (37%, 34–39).^7^ The WHO recognises sepsis as a global health priority in the coming decade,^8^ with the highest incidence of sepsis among neonates and young children. New solutions to address serious infections in young children are needed in order to achieve Sustainable Development Goal 3.2 to ‘End preventable deaths of newborns and children under age five years of age with all countries aiming to reduce neonatal mortality to at least as low as 12 per 1000 live births and under-five mortality to at least as low as 25 per 1000 live births’.^9^

Data are scarce on risk factors and aetiology of community-acquired serious infections in developing countries.^10–21^ Laboratory confirmation of the aetiology of bacterial infections is particularly lacking in community (non-healthcare) settings where most infections take place. Empirical antibiotic treatment for a constellation of neonatal sepsis symptoms predominates^21–25^ and treatment regimens vary both between and within regions.^24^ Meanwhile, the use of neonatal risk factor scoring for guiding management decisions for possible serious bacterial infections (pSBI) has shown utility for improving antibiotic stewardship and reducing neonatal early-onset sepsis-based mortality.^26–31^ These approaches, however, are largely based on symptoms in hospitalised infants. High-quality community-based data regarding risk factors for infections may serve to inform preventive policies and clinical practices surrounding neonatal sepsis; possibly including improved scoring algorithms.

The Aetiology of Neonatal Infection in South Asia (ANISA) study was designed^32–34^ to increase understanding of factors that predispose young infants to, or protect them from, infections at the community level and to provide data to inform evidence-based strategies to reduce neonatal infections and mortality. Here we use data from five diverse community-based urban and rural ANISA sites to explore risk factors predisposing young infants in South Asia to contracting serious infections attributable to bacterial pathogens.^34^

## Methods

ANISA was a longitudinal community-based prospective cohort study of mother–infant pairs drawn from five population-based sites in Bangladesh, India and Pakistan from 2011 to 2014. Study site characteristics and sepsis surveillance methodologies have been described elsewhere.^32^ In brief, active pregnancy surveillance was established by community health workers (CHWs) who registered all consenting married women of reproductive age in each catchment area. CHWs and study physicians collected a variety of information from mothers using standardised questionnaires administered during the earliest stages of pregnancy, shortly after childbirth and during 59-day longitudinal follow-up of infants. Data were collected on risk factors including participant demographics, home environment, pregnancy and birth history, postpartum maternal characteristics and neonatal characteristics at birth (see web online supplemental tables 1–5). CHWs visited mothers and newborns up to 10 times, thrice in the first week after birth and weekly thereafter until 59 days after birth. Infants presenting with one or more of seven clinical signs of pSBI were referred to study physicians. The signs used to identify pSBI included feeding poorly or not feeding at all, no movement at all or movement only when stimulated, fast breathing (≥60 breaths/min), elevated temperature (>38°C), hypothermia (<35.5°C), chest in-drawing and convulsions.^31 32^ On physician diagnosis of pSBI, blood specimens were taken for conventional blood culture and blood and nasopharyngeal (NP) swab specimens were taken for multipathogen quantitative real-time PCR (qPCR) analysis; detailed methods to determine aetiologies of infection are described elsewhere^2 33^ and these data are used in this analysis. Age-matched and site-matched control newborn infants were identified^35^ and were assessed by study physicians to rule out the presence of clinical signs of pSBI. Blood and NP swab samples were collected to identify the presence of pathogen-derived nucleic acid in non-symptomatic control infants using qPCR; conventional blood culture was not performed on control specimens.^33 35^ These control specimens were used to inform the false positivity rate in the Partial Latent Class Model (PLCM). Quality of field surveillance and laboratory procedures and processes was maintained by consistent monitoring of field and laboratory operations and tracking of specimens using a purpose-built digital tracking system at all sites as described previously.^36^

10.1136/bmjgh-2022-009706.supp1 Supplementary data

### Patient and public involvement

The study was developed together with multidisciplinary teams based at each of the five study sites; a common protocol and questionnaire was developed and customised for use in the local languages and circumstances. CHWs were recruited from the local communities but patients (mother–child pairs) were not directly involved in the design of the questionnaires, study recruitment, diagnostic, scientific or statistical methods used to analyse their data. Findings will be disseminated by the local partners to clinics that serve the communities, and findings may be more broadly shared in their respective regions by the study site teams.

### Statistical analysis

The risk factor analysis reported here directly used the ANISA Bayesian PLCM attribution data that have been reported previously. Wherein each young infant with pSBI (based on the presence of one or more of seven danger signs) underwent blood culture and both blood and respiratory qPCR assays; pathogen(s) may have been detected in one or more of these tests. Blood culture results were further characterised as definite pathogens versus contaminants using an expert panel that systematically reviewed clinical and diagnostic information on each case.^37^ In order to properly attribute a particular child’s pSBI to a specific pathogen type, the output from each of these analyses were integrated into a PLCM combining the multiple tests along with their different error rates as described previously.^2 38 39^ The output of this model was mean pathogen proportions of 28 target pathogens and two additional classes: other blood culture (all organisms isolated from blood culture that did not have a matching molecular assay test) and other/none (if no aetiology was attributed). These proportions were performed at individual level with 95% credible intervals (CrIs). Performance of this modelling was assessed by internal simulation studies; model convergence was assessed through trace and other diagnostic plots. The programming and computation of the PLCM was performed with R (V3.2.5), SAS (V.9.3) and Stata (V.13.1) and was reported previously.^2^

For the risk factor analysis reported here, 50 complete PLCM output sets were selected at random from a set of 2000 complete, stable, PLCM output runs; inclusion of additional sets was not found to stabilise or notably alter risk factor output. These iterations were then further randomly resorted for each child to produce 50 synthetic model outputs for analysis.

Multiple imputation function was used to perform and combine analyses of these randomised 50 model output sets against each of the potential risk factors using stepwise logistic multivariable multiple regression modelling; multiple imputation allowed for combining the resulting point estimates from 50 runs to account for the uncertainty in the outcome in the intervals around the given risk factor estimates.^40^ Multiple imputation was not used to correct for incomplete data in the underlying risk factor dataset, but rather to combine the PLCM estimates of pathogen attribution and given risk factor into a single estimate and variance, incorporating both the within-imputation and between-imputation variability.^40^ Bivariate analysis on each putative risk factor’s impact on bacterial infection was performed first. Using these outputs, a multivariable regression model was constructed within each of five predefined risk domains (neonatal, maternal, birth procedure, environmental and demographic factors), controlling for site. Nested, circular variables such as duration of hospitalisation after birth and whether a young infant was reportedly ever hospitalised were removed from multivariable modelling. Using stepwise elimination of non-significant factors, the remaining significant factors in each risk domain were then combined into the final risk model. Risk factors with p value ≤0.10 and relative risk (RR) values either above 1.1, signifying risk, or below 0.9090, signifying protection, were considered as having statistical significance. Models were then combined in the following order: neonatal health factors, maternal factors, birth procedure factors, environmental factors and finally demographic factors. This analysis was performed in Stata (V.13.1 SE).

Low birth weight (LBW) was defined as 1500–2500 g and very LBW (VLBW) as <1500 g. Nutritional risk status of mothers was defined by mid-upper-arm circumference, with high nutritional risk <20.7 cm and moderate nutritional risk <23 cm.^41^ Moderate/late preterm birth was defined as birth at 32–37 weeks’ gestation, and very and extremely preterm was defined as <32 weeks’ gestation. Premature rupture of membranes (PROM) was defined as water breaking before onset of labour pain. The country-specific wealth index and household wealth quintiles were calculated using information on durable household assets, construction materials, utilities, etc.^42 43^

## Results

### Population characteristics

**Figure 1:**
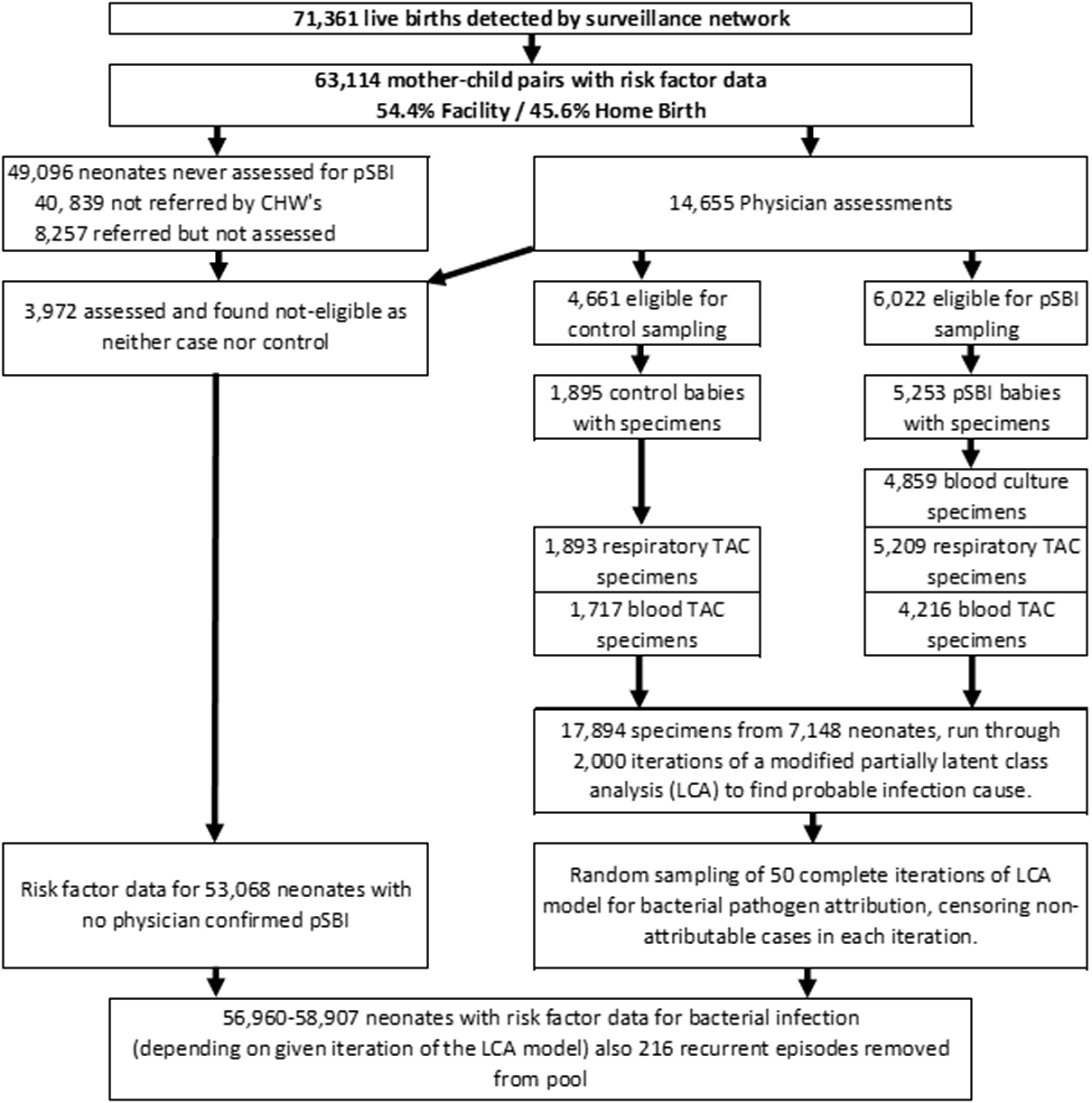
Characteristics of the infants enrolled and analysed in the ANISA study. ANISA, Aetiology of Neonatal Infection in South Asia; CHWs, community health workers; pSBI, possible serious bacterial infection; TAC, TaqMan® Array Card

Between November 2011 and March 2014, we enrolled 63 114 newborn–mother pairs and followed them until the infants were 2 months old (figure 1); 73.9% (n=46 673) of the newborns were registered within 24 hours of birth and the rest (26.1%, n=16 441) between their second and seventh days after birth. Among the registered newborns, 51% (n=32 419) were male, 27% (n=16 832) were born with LBW and 19% (n=11 837) were preterm. Mothers of 47.3% (n=29 840) of the infants attended at least four antenatal care visits, 63.7% (n=40 186) of the deliveries took place in healthcare facilities and 67.6% (n=42 669) of infants received colostrum. There were 6022 pSBI episodes identified by study physicians, resulting in the collection and analysis of 4859 blood specimens (80.6% of those indicated) and 5209 respiratory specimens (86.5%). We also collected 1717 blood specimens and 1893 respiratory specimens from 4661 age-matched and site-matched healthy control participants.

### Neonatal health status factors

We analysed eight neonatal factors, five of which were significantly associated with bacterial infection in bivariate analysis (online supplemental table 1). Multivariable analysis of neonatal factors alone, controlling for site, showed that LBW (RR 1.76, 95% CrI 1.45 to 2.13), VLBW (RR 6.20, 95% CrI 4.05 to 9.51), male sex (RR 1.27, 95% CrI 1.07 to 1.52) and problems with breathing initiation at birth (RR 2.54, 95% CrI 2.01 to 3.21) were associated with increased risk of bacterial infections (table 1).

**Table 1:** Neonatal risk factors multivariate model – all sites

	Bacterial infection		
	Relative risk	P value	95% CrI
Sylhet, Bangladesh	1.00		
Karachi, Pakistan	1.23	0.110	0.95 to 1.60
Matiari, Pakistan	0.86	0.263	0.65 to 1.12
Vellore, India	1.35	0.098	0.95 to 1.91
Odisha, India	2.81	0.000***	2.17 to 3.63
Birth weight (normal birth weight >2500 g baseline)			
Low birth weight <2500 g	1.78	0.000***	1.47 to 2.16
Very low birth weight <1500 g	6.19	0.000***	4.03 to 9.51
Sex			
Male	1.28	0.006**	1.07 to 1.53
Breathing initiation problems at birth	2.55	0.000***	2.01 to 3.24
Constant	0.01	0.000***	0.01 to 0.01
Sample size variation	n=56 960–57 038		

Exponentiated coefficients.

*P<0.05, **p<0.01, ***p<0.001.

CrI, credible interval.

### Maternal factors

We analysed 24 maternal risk factors, and six were found to be associated with the development of bacterial infections in bivariate analysis (online supplemental table 2). Multivariable analysis of maternal factors alone, while controlling for site, revealed that having one (RR 0.73, 95% CrI 0.58 to 0.92) or two (RR 0.68, 95% CrI 0.51 to 0.92) prior births and having four or more antenatal care visits (RR 0.74, 95% CrI 0.57 to 0.96) were significantly protective against risk of bacterial infection (table 2).

**Table 2:** Maternal risk factors multivariate model – all sites

	Bacterial infection		
	Relative risk	P value	95% CrI
Sylhet, Bangladesh	1.00		
Karachi, Pakistan	1.17	0.267	0.89 to 1.54
Matiari, Pakistan	1.05	0.734	0.79 to 1.40
Vellore, India	1.46	0.094	0.94 to 2.28
Odisha, India	2.47	0.000***	1.88 to 3.24
Prior children			
No prior children			
1 child	0.73	0.008**	0.58 to 0.92
2 children	0.68	0.012*	0.51 to 0.92
3+ children	0.94	0.613	0.75 to 1.18
Antenatal care visits (ANC)			
No ANC visits			
1 ANC visit	0.83	0.261	0.59 to 1.15
2 ANC visits	0.94	0.692	0.69 to 1.28
3 ANC visits	0.96	0.774	0.71 to 1.29
4+ ANC visits	0.74	0.024*	0.57 to 0.96
Constant	0.01	0.000***	0.01 to 0.02
Sample size variation	n=57 974–58 058		

Exponentiated coefficients.

*P<0.05, **p<0.01, ***p<0.001.

CrI, credible interval.

### Birth procedure risk factors

Nineteen risk factors surrounding the birth event were analysed; six factors related to birth procedures and behaviours of the birth team were significant (online supplemental table 3). Multivariable analysis of birth procedure factors showed that presenting with PROM (RR 1.29, 95% CrI 1.04 to 1.60) was the only birth-procedure related factor that increased risk of bacterial infection (table 3).

**Table 3:** Birth procedure risk factor multivariate model – all sites

	Bacterial infection		
	Relative risk	P value	95% CrI
Sylhet, Bangladesh	1.00		
Karachi, Pakistan	1.22	0.13	0.94 to 1.58
Matiari, Pakistan	0.99	0.94	0.76 to 1.30
Vellore, India	1.01	0.95	0.71 to 1.44
Odisha, India	2.16	0.000***	1.67 to 2.79
Premature rupture of membranes	1.29	0.019*	1.04 to 1.60
Constant	0.01	0.000***	0.01 to 0.01
Sample size variation	n=58 822–58 906		

Exponentiated coefficients.

*P<0.05, **p<0.01, ***p<0.001.

CrI, credible interval.

### Environmental factors

Twelve risks were analysed related to the immediate environment encountered following birth. Two factors were found to be independently significant (online supplemental table 4). Multivariable analysis of environmental factors showed that having unsanitary or no formal toilet facilities (RR 1.32, 95% CrI 1.01 to 1.71) and lack of household hand washing facilities (RR 1.34, 95% CrI 1.03 to 1.74) increased the risk of bacterial infections. Also, being situated in Odisha, India (RR 2.71, 95% CrI 1.97 to 3.74) and Karachi, Pakistan (RR 1.57, 95% CrI 1.15 to 2.15) showed significantly increased risk compared with Sylhet, Bangladesh (table 4).

**Table 4:** Environmental risk factors multivariate model – all sites

	Bacterial infection		
	Relative risk	P value	95% CrI
Sylhet, Bangladesh	1.00		
Karachi, Pakistan	1.57	0.005**	1.15 to 2.15
Matiari, Pakistan	1.24	0.154	0.92 to 1.66
Vellore, India	1.22	0.308	0.83 to 1.81
Odisha, India	2.71	0.000***	1.97 to 3.74
Toilet facilities			
Flush toilet	1.00		
Latrine/VIP latrine	1.32	0.041*	1.01 to 1.71
Unsanitary or no formal Toilet	1.16	0.213	0.92 to 1.48
Unknown	0.75	0.647	0.22 to 2.56
No hand washing facilities in home	1.34	0.028*	1.03 to 1.74
Constant	0.01	0.000***	0.01 to 0.01
Sample size variation	n=58 823 –58 907		

Exponentiated coefficients.

*P<0.05, **p<0.01, ***p<0.001.

CrI, credible interval.

### Demographic factors

Five demographic risk factors were analysed related to parental education, maternal work and decision making in the household (online supplemental table 5). Only socioeconomic status (SES) was found to be independently significant. Bacterial infection risk was significantly elevated in Odisha (RR 2.32, 95% CrI 1.82 to 2.97) when compared with Sylhet. Multivariable analysis of demographic factors revealed that only low SES was associated with significantly elevated risk of bacterial infection in the first (RR 1.78, 95% CrI 1.35 to 2.36), second (RR 1.64, 95% CrI 1.24 to 2.19) and third (RR 1.62, 95% CrI 1.20 to 2.17) wealth quintiles when compared with the fifth (wealthiest) SES quintile (table 5).

**Table 5:** Demographic risk factors multivariate model – all sites

	Bacterial infection		
	Relative risk	P value	95% CrI
Sylhet, Bangladesh	1.00		
Karachi, Pakistan	1.20	0.152	0.93 to 1.55
Matiari, Pakistan	1.10	0.471	0.85 to 1.43
Vellore, India	1.03	0.858	0.73 to1.45
Odisha, India	2.32	0.000***	1.82 to 2.97
Socioeconomic status			
Fifth quintile (highest)			
Fourth quintile	1.33	0.068	0.98 to 1.80
Third quintile	1.62	0.001***	1.20 to 2.17
Second quintile	1.64	0.001***	1.24 to 2.19
First quintile (lowest)	1.78	0.000***	1.35 to 2.36
Constant	0.01	0.000***	0.01 to 0.01
Sample size variation	n=58 368–58 452		

Exponentiated coefficients.

*P<0.05, **p<0.01, ***p<0.001.

CrI, credible interval.

### Full multivariable model results

Pooled multivariable analysis showed that VLBW (<1500 g) increased risk of bacterial infection more than fivefold (RR 5.77, 95% CrI 3.73 to 8.94), and LBW (<1500–2500 g) remained a significantly elevated risk factor (RR 1.73, 95% CrI 1.42 to 2.11). Breathing initiation problems at birth (RR 2.50, 95% CrI 1.96 to 3.18) and male sex (RR 1.27, 95% CrI 1.07 to 1.52) also were associated with increased risk of bacterial infection (table 6). PROM was also a significant risk factor in the final model (RR 1.27, 95% CrI 1.03 to 1.58). Having one or two prior children, as well as a non-flush toilet and lack of hand washing facilities all lost significance with the addition of SES into the overall model. Being part of the lowest three SES quintiles, compared with the top/wealthiest quintile, remained a significant risk factor in the final model (first RR 1.52, 95% CrI 1.07 to 2.16; second RR 1.41, 95% CrI 1.00 to 1.97; third RR 1.42, 95% CrI 1.01 to 1.99).

**Table 6:** Overall multivariate bacterial infection risk factor model

	Neonatal factors		+ Maternal factors		+ Birth procedure factors		+ Environmental factors		+ Demographic factors	
	Relative risk	95% CrI	Relative risk	95% CrI	Relative risk	95% CrI	Relative risk	95% CrI	Relative risk	95% CrI
Sylhet, Bangladesh	1.00	.	1.00	.	1.00	.	1.00	.	1.00	.
Karachi, Pakistan	1.23	0.95 to 1.60	1.28*	0.98 to 1.68	1.29	0.98 to 1.69	1.62**	1.18 to 2.26	1.49*	1.06 to 2.09
Matiari, Pakistan	0.86	0.65 to 1.12	0.87	0.66 to 1.15	0.81	0.61 to 1.08	0.91	0.67 to 1.24	0.92	0.67 to 1.25
Vellore, India	1.35*	0.95 to 1.91	1.65*	1.11 to 2.47	1.67*	1.12 to 2.50	1.96**	1.26 to 3.04	1.84*	1.18 to 2.88
Odisha, India	2.81***	2.17 to 3.63	3.21***	2.42 to 4.26	3.06***	2.30 to 4.08	3.47***	2.44 to 4.94	3.47***	2.42 to 4.96
Birth weight (>2500 g baseline)										
Low birth weight <2500 g	1.78***	1.47 to 2.16	1.78***	1.46 to 2.17	1.78***	1.46 to 2.16	1.76***	1.45 to 2.15	1.73***	1.42 to 2.11
Very low birth weight <1500 g	6.19***	4.03 to 9.51	6.25***	4.07 to 9.60	6.24***	4.07 to 9.58	6.11***	3.98 to 9.39	5.77***	3.73 to 8.94
Male sex	1.28**	1.07 to 1.53	1.27**	1.07 to 1.52	1.27**	1.07 to 1.52	1.27**	1.07 to 1.52	1.27**	1.07 to 1.52
Breathing problems at birth	2.55***	2.01 to 3.24	2.55***	2.01 to 3.24	2.53***	1.99 to 3.22	2.51***	1.98 to 3.20	2.50***	1.96 to 3.18
Antenatal care visits (ANCs) (no visits baseline)										
1 ANC visit			0.81	0.58 to 1.14	0.82*	0.58 to 1.15	0.85	0.60 to 1.19	0.86	0.61 to 1.21
2 ANC visits			0.95	0.69 to 1.29	0.94	0.69 to 1.29	0.97	0.71 to 1.33	0.99	0.72 to 1.37
3 ANC visits			1.00	0.74 to 1.36	0.99	0.73 to 1.35	1.03	0.76 to 1.40	1.05	0.77 to 1.44
4+ ANC visits			0.79*	0.60 to 1.04	0.79*	0.60 to 1.03	0.83	0.63 to 1.09	0.88	0.66 to 1.17
Prior children (no prior children baseline)										
1 child			0.81	0.64 to 1.02	0.81*	0.64 to 1.03	0.81*	0.64 to 1.02	0.80	0.63 to 1.02
2 children			0.78	0.58 to 1.05	0.78	0.58 to 1.05	0.77*	0.57 to 1.04	0.76	0.56 to 1.02
3+ children			1.09	0.86 to 1.37	1.09	0.86 to 1.38	1.07	0.84 to 1.35	1.03	0.81 to 1.32
PROM					1.26*	1.02 to 1.57	1.26*	1.02 to 1.56	1.27*	1.03 to 1.58
Toilet facilities (flush toilet baseline)										
Latrine/VIP latrine							1.27*	0.97 to 1.66	1.15	0.86 to 1.53
Unsanitary or no formal toilet							1.09	0.85 to 1.40	0.94	0.71 to 1.25
No handwashing station in home							1.28*	0.98 to 1.67	1.21	0.92 to 1.58
Socioeconomic status (fifth/top quintile baseline)										
Fourth quintile									1.26	0.92 to 1.75
Third quintile									1.42*	1.01 to 1.99
Second quintile									1.41*	1.00 to 1.97
First (bottom) quintile									1.52*	1.07 to 2.16
Constant	0.01***	0.01 to 0.01	0.02	0.01 to 0.01***	0.01***	0.01 to 0.01	0.01***	0.00 to 0.01	0.00***	0.00 to 0.01
Sample variation across imputations	56 960 to 57 038		56 147 to 56 229		56 146 to 56 228		56 146 to 56 228		55 719 to 55 801	

Exponentiated coefficients.

*P<0.10, **p<0.01, ***p<0.001.

CrI, credible interval; PROM, premature rupture of membrane.

## Discussion

This analysis, through examination of risk factors in rural and urban communities across five sites in three countries, found that a small set of factors—VLBW/LBW, male sex, PROM and breathing problems at birth and SES—were significantly associated with laboratory-confirmed bacterial infections. These factors can be directly observed at birth, except for SES. These findings are congruent with simplified clinical decision making and may support development of algorithms that can be used to improve antibiotic stewardship in the Indian subcontinent. Taking clinical presentation of risk factors into decision making could allow for improved targeting of scarce resources and antibiotic therapy. Moreover, these findings suggest that aggressive strategies that combat poverty are important in reducing risk for bacterial infections.

Results from this analysis are consistent with other studies—including a recent systematic review and meta-analysis of 15 studies in India—that found that risk factors for neonatal sepsis included low gestational age, male sex, breathing problems at birth and PROM, among other factors such as home birth.^19 20 44^ Some results from this analysis were surprising, particularly that several reported birth procedure-related risk factors lost significance in multivariable analysis; non-facility/home birth, lack of use of a non-sterile instrument or materials to cut and tie the umbilicus were significant bivariate factors but lost significance in multivariable modelling. Similarly, maternal antenatal care and unsanitary or no formal toilet facilities and lack of household hand washing facilities lost significance in multivariable modelling. Other factors, such as frequent vaginal exams, the presence of skilled birth attendants, birth attendant hand washing and feeding of colostrum were not found to be significant risk factors, even during bivariate analysis. Other studies had previously identified unhygienic intrapartum and postnatal care, poor prelacteal feeding and contaminated foods and fluids as important contributors to the pathogenesis of neonatal infection and priorities in prevention of newborn infections.^44 45^

Male children have been shown to have a greater biological risk than females who often have heightened social risks for infection later in life.^46–48^ Future analysis stratifying by sex could add insight and contribute to corrective strategies. A similar risk factor analysis on viral aetiology within the ANISA study is forthcoming, along with other important related risk analyses, such as aetiology-specific (Respiratory Syncytial Virus, RSV & Group B Streptococcus, GBS), recurrent infections and mortality.

Over the past decades, a variety of management guidelines for neonatal infections have been developed to manage newborn infants with suspected infections. These guidelines were deliberately based on high sensitivity in identifying potential cases, leading to overtreatment and potentially contributing to concomitant high rates of antimicrobial resistance (AMR).^49 50^ Although community-based use of antibiotics for pSBI has been shown to reduce neonatal mortality,^23 51–55^ the fear of missing a potentially treatable infection leads to overprescription of broad-spectrum empirical antibiotics with the attendant potential for side effects and propagation of AMR.^56^ Our Bayesian PLCM analysis directly used the heterogeneous samples and test outputs from the ANISA study pathogen attribution. It used the true error rates to assign relative risks associated with bacterial infection in South Asia.

These findings provide important insights into those factors which, if addressed, have potential to target bacterial sepsis cases, reducing cost, morbidities and case fatalities among infants across the subcontinent and beyond. Importantly, most of these factors are readily detectable at birth, allowing early intervention using targeted protocols and training in the community setting. Improving targeting of empiric antibiotic treatments to the most at-risk infants early has the potential to improve case management, reduce morbidity and mortality and limit unnecessary empirical antibiotic treatment and potential emergence of AMR.

### Limitations

Despite testing of both blood and respiratory samples, prior analysis showed that no cause could be attributed to 72% of pSBI episodes and only 11% of infants who died had samples taken within 7 days of death.^2^ This impacts the availability of pathogen data in the underlying attribution model, which feeds into this risk analysis. However, it should be noted that the number of attributable infections was nearly double among those babies who died compared with those who did not and more than 90% of those were bacterial infections and are thus included in this model. Design of new initiatives has already begun to fill this knowledge gap.^57^

For young children who were either born in medical facilities and stayed for long periods due to the detection of clinical symptoms at birth or neonates who contracted a nosocomial infection while in medical facilities and were kept for treatment, the risk factors collected regarding length of hospital stay became highly circular. This made it difficult to parse whether the hospital stay was the source of the infection (nosocomial) or hospitalisation was due to the community acquisition of the infection. Therefore, risk presented by ‘time spent in hospital’, although a major factor in risk for infection, was removed from multivariable analyses to avoid this bias in favour of community-based acquisition of infections (online supplemental table 3).

Specific timing of onset of symptoms, treatment course/duration, coinfections and other clinical factors were not considered in this aggregate analysis. Data on Apgar scores and caesarean section status were not collected, precluding comparison on these variables with studies that collected these data.

## Conclusion

Motivated by the increasing proportion of neonatal deaths among under-five deaths, ANISA represents the largest community-based study of its kind, bridging the knowledge gap in aetiology of neonatal sepsis using intensive and early surveillance, state-of-the-art laboratory methods, appropriate controls and sophisticated modelling. This analysis provides important insights into the risk factors for neonatal sepsis in South Asia: a region where the preponderance of the world’s neonatal infections and deaths occur.^58 59^ Since all analyses were adjusted by site, this helps to control confounding in any given setting in the study population.

Risk factor data collected by ANISA could potentially be used to develop point-of-care algorithms to better target infants with high likelihood of bacterial infections in order to target simplified generalised antibiotic regimens employing conventional community-based strategies and/or data-driven risk scoring algorithms.^28 29 60^ If validated, such algorithms could be used to help ensure appropriate treatment of neonates at high risk for bacterial infection and limit unnecessary antibiotic use among neonates in whom bacterial infection is unlikely.

## Data Availability

Data may be obtained from a third party and are not publicly available. The data collected as part of the ANISA study were shared with all participating collaborators but will not be made available to others.
