# Supplementary material for: Risk factors for community-acquired bacterial infection among young infants in South Asia: a longitudinal cohort study with nested case–control analysis
Source: BMJ Glob Health. 2022 Nov 1;7(11):e009706. doi: 10.1136/bmjgh-2022-009706 (PMC9628539; doi:10.1136/bmjgh-2022-009706)
Supplement: Supplementary data [file bmjgh-2022-009706supp001.pdf]

Web Tables for: Risk factors for community-acquired bacterial infection among South Asian young infants: a primary analysis of the ANISA longitudinal cohort study with nested case-–control analysis

Web Table 1 - Neonatal health status risk factors – univariate analysis

	Bacterial Infection		
	**Relative Risk**	**p-value**	**95% Cred. Int**
Moderate or late preterm (32- <37 weeks)	1.34	0.013*	1.06,1.68
Very and extremely preterm (<32 weeks)	1.72	0.026*	1.07,2.77
Multiple births (pluripara)	1.82	0.018*	1.12,2.98
Low birth weight <2500g	1.66	0.000***	1.37,2.00
Very low birth weight <1500g	5.83	0.000***	3.86,8.82
Male sex	1.26	0.010*	1.06,1.50
Congenital anomaly detected at birth	2.41	0.076	0.91,6.39
Breathing problems reported at birth	2.18	0.000***	1.75,2.70
Jaundice (mild and moderate)*	0.87	0.490	0.59,1.29
Severe jaundice*	2.17	0.116	0.83,5.69
Skin pustules detected	0.60	0.537	0.12,3.05
Sample size variation	n = 57,353 - 58,907		

*Physician reported

Web Table 2 - Maternal risk factors – univariate analysis

	Bacterial Infection		
	**Relative Risk**	**p-value**	**95% Cred. Int**
Birth spacing 18-59 months from last birth	0.79	0.007**	0.67,0.94
Birth spacing < 18 months from last birth	0.71	0.079	0.48,1.04
Prior births			
1 Prior birth	0.74	0.010	0.59,0.93
2 Prior births	0.62	0.002**	0.46,0.83
3+ Prior births	0.84	0.078	0.69,1.02
Maternal fever reported - prior 7 days	1.19	0.293	0.86,1.63
Fever during pregnancy	1.20	0.167	0.93,1.56
Foul discharge reported - pregnancy	1.27	0.322	0.79,2.06
Foul discharge reported - birth	1.21	0.129	0.95,1.53
Bleeding reported during pregnancy	1.35	0.400	0.67,2.72
Convulsions reported during pregnancy	1.43	0.516	0.49,4.18
Swelling reported during pregnancy	0.93	0.576	0.73,1.19
Moderate nutritional risk (MUAC) (<23cm)	1.02	0.872	0.82,1.27
High nutritional risk (MUAC) (<20.7cm)	0.98	0.923	0.63,1.52
Unknown nutritional status	1.16	0.282	0.88,1.52
Physical work reported during pregnancy	1.18	0.074	0.98,1.42
Respiratory illness reported	1.11	0.404	0.87,1.41
Antenatal care (ANC) visits (none baseline)			
1 ANC visit	1.04	0.814	0.75,1.44
2 ANC visits	1.06	0.698	0.79,1.44
3 ANC visits	1.16	0.315	0.87,1.54
4+ ANC visits	0.95	0.638	0.75,1.19
One TT** dose during this pregnancy	0.94	0.692	0.70,1.27

Two TT** doses during this pregnancy	1.22	0.035*	1.01,1.46
1 TT dose over lifetime	0.90	0.639	0.58,1.39
2+ TT doses over lifetime	0.98	0.832	0.79,1.21
Iron folate supplementation (0-2 months baseline)			
2-5 months	1.00	0.974	0.79,1.28
6+ months	0.67	0.025*	0.47,0.95
Do not know	1.00	0.983	0.76,1.33
Maternal tobacco use	1.15	0.711	0.56,2.34
Cooking smoke during pregnancy	0.98	0.948	0.61,1.60
Mother uses paan / betal leaf	1.00	0.985	0.81,1.24
Sample size variation		n = 44,738 - 58,907	

* Mid-Upper Arm Circumference (MUAC)

**Tetanus Toxoid (TT) Vaccine injection

Web Table 3 Birth procedure-related risk factors – Unadjusted values

	Bacterial Infection		
	**Relative Risk**	**p-value**	**95% Cred. Int**
Non-skilled birth attendant	0.86	0.088	0.72,1.02
Sterile instrument* used to cut umbilical cord	1	.	.,.
Non-sterile instrument used to cut umbilical cord	1.21	0.039*	1.01,1.44
Unknown implement used to cut umbilical cord	0.99	0.973	0.61,1.62
No clean delivery kit used	0.78	0.014*	0.634,0.95
Hand washing	1	.	.,.
Birth attendant did not wash hands at delivery	0.80	0.406	0.47,1.36
Other caregiver(s) did not wash hands	1.16	0.401	0.82,1.66
Vaginal examinations (none performed baseline)			
1 Exam	0.88	0.421	0.64,1.21
2 Exams	1.09	0.424	0.88,1.36
3 or more exams	1.02	0.866	0.78,1.34
Unknown	1.17	0.581	0.67,2.04
Non-clear amniotic fluid	1.13	0.382	0.86,1.47
Duration of labor (baseline 0-6 hours)			
6-11 hours	1.11	0.358	0.89,1.38
12-23 hours	1.41	0.005**	1.11,1.80
24-47 hours	1.11	0.602	0.75,1.64
48-71 hours	1.22	0.728	0.39,3.78
72+ hours	0.18	0.995	0.00,1.38 $^{e+216}$
Duration of water break to birth (0-6 hours baseline)			
6-11 hours	1.39	0.042*	1.013,1.91
12-23 hours	1.77	0.000***	1.29,2.42
24-47 hours	2.04	0.000***	1.40,2.97
48-71 hours	1.70	0.214	0.74,3.87
72+ hours	0.56	0.994	0.00,2.16^{e+67}
Duration of Stay in Facility (No stay nominal value)			
<24 hours in health facility	9.40	0.000***	7.66,11.54
24 hours - <48 hours in health facility	9.73	0.000***	6.75,14.04
48 hours - < 72 hours in health facility	6.50	0.000***	4.12,10.24
>72 hours in health facility	0.25	0.000***	0.19,0.34
PROM (water broke before labor pain)	1.37	0.003**	1.11,1.68

Birth position (lying on covered floor baseline)			
Lying on bare (uncovered) floor	1.38	0.309	0.74,2.58
Lying on bed	1.26	0.015*	1.05,1.52
Squatting	1.28	0.439	0.68,2.39
Delivery attendant			
Health worker	0.96	0.654	0.79,1.16
Unknown	1.33	0.643	0.39,4.52
Vernix left on after birth	1	.	.,.
Vernix cleaned after birth	1.08	0.429	0.90,1.29
Unknown	1.38	0.280	0.77,2.47
Wrapped < 10 minutes	1	.	.,.
Wrapped 10-30 minutes	0.98	0.867	0.77,1.24
Wrapped 31-60 minutes	1.71	0.164	0.80,3.65
Wrapped > 60 minutes	1.76	0.054	0.99,3.12
Not wrapped/unknown	0.63	0.161	0.33,1.21
Unclean umbilical binding	1.37	0.001***	1.14,1.64
Sterile substance** applied	1	.	.,.
No substance applied	0.99	0.957	0.71,1.38
Non-sterile substance applied	0.81	0.291	0.55,1.20
Unknown	1.46	0.180	0.84,2.53
Colostrum given	1	.	.,.
Colostrum discarded	0.83	0.101	0.67,1.04
Unknown	2.10	0.000***	1.64,2.69
Exclusive breastfeeding	1	.	.,.
Non-exclusive breastfeeding	0.96	0.661	0.78,1.17
Non-facility delivery	0.83	0.033*	0.69,0.98
No reported stay in hospital	1	.	.,.
Stay in hospital 1 day or less	5.38	0.000***	3.80,7.60
Stay in hospital over 1 day	1.81	0.220	0.70,4.64
Sample size variation		n = 55,168-58,907	

Exponentiated coefficients: * p < 0.05, ** p < 0.01, *** p < 0.001
***Sterile instrument: sterile delivery kit, unused blade.**
****Sterile Substance applied: antiseptic or antibiotic substance.**

Web Table 4 - Environmental risk factors – Unadjusted values

	Bacterial Infection		
	Relative Risk	p-value	95% Cred. Int
Respiratory symptoms reported in household	0.99	0.916	0.69,1.40
One or more under-five children	0.78	0.006**	0.65,0.93
Water from a unclean source	0.82	0.142	0.63,1.07
Toilet facilities (flush baseline)			
Latrine/VIP latrine	1.11	0.328	0.90,1.37
Unsanitary or no formal toilet	1.51	0.000***	1.22,1.86
No handwashing station present in home	1.24	0.091	0.97,1.59
Goats, cows, buffaloes live in the house or compound	0.98	0.832	0.83,1.17
Hen, chicken, ducks, geese live in the house or compound	1.14	0.164	0.95,1.37
Other birds live in the house or compound	1.27	0.359	0.76,2.10
Dogs live in the house or compound	1.08	0.535	0.85,1.36
Cats live in the house or compound	1.06	0.599	0.85,1.33
Other animals live in the house or compound	1.25	0.718	0.37,4.27
Any animals in the compound	0.98	0.864	0.81,1.19
Sample size variation	n = 58,370-59,687		

Exponentiated coefficients: * p < 0.05, ** p < 0.01, *** p < 0.001

Web Table 5 - Demographic risk factors – Unadjusted values

	Bacterial Infection		
	Relative Risk	p-value	95% Cred. Int
**Socio-economic status*** (Highest SES quintile baseline)			
Fourth SES quintile	1.32	0.070	0.98,1.79
Third SES quintile	1.62	0.001**	1.21,2.17
Second SES quintile	1.66	0.001***	1.25,2.20
First (lowest) SES quintile	1.81	0.000***	1.36,2.39
**Maternal Education** (No education baseline)			
Primary incomplete	1.05	0.808	0.73,1.49
Primary complete	1.05	0.741	0.80,1.37
Secondary incomplete	0.82	0.116	0.64,1.05
Above secondary	0.96	0.746	0.75,1.23
**Paternal Education** (No education baseline)			
Primary incomplete	1.14	0.453	0.81,1.61
Primary complete	1.00	0.975	0.76,1.33
Secondary incomplete	1.06	0.670	0.82,1.36
Above secondary	0.93	0.531	0.75,1.16
**Maternal Work** (No work baseline)			
Works in home	1.11	0.406	0.87,1.43
Works outside home	1.08	0.691	0.73,1.61
**Maternal Decision Making**			
Mother not involved in major purchasing decisions	0.91	0.349	0.75,1.11
Mother not involved in household purchase decisions	0.94	0.527	0.78,1.14
Mother not involved in visitation decisions	0.88	0.158	0.73,1.05
Mother not involved in health decisions	0.85	0.074	0.71,1.02
Sample size variation	n = 58,368-58,907		

Exponentiated coefficients: * p < 0.05, ** p < 0.01, *** p < 0.001
*** SES based on site-specific wealth quintile**
